# Tuberous sclerosis complex associated lymphangioleiomyomatosis caused by de novo mutation of *TSC2* gene in Vietnam: A case report

**DOI:** 10.1002/rcr2.1346

**Published:** 2024-04-09

**Authors:** Dinh Van Luong, Le Ngoc Huy, Nguyen Xuan Giang, Nguyen Huu Hong Thu, Nguyen Hai Ha, Nguyen Huy Binh

**Affiliations:** ^1^ Lung Transplant Center National Lung Hospital Hanoi Vietnam; ^2^ Tuberculosis and Lung Diseases Department Hanoi Medical University Hanoi Vietnam; ^3^ Institute of Genome Research Vietnam Academy of Science and Technology Hanoi Vietnam; ^4^ Physiology Department Hanoi Medical University Hanoi Vietnam

**Keywords:** pathogenic variants, *TSC2* gene, TSC‐LAM

## Abstract

Lymphangioleiomyomatosis (LAM) represents a rare, insidiously progressive disease of the pulmonary system, marked by cystic degradation of lung tissues leading to respiratory compromise. Pulmonary LAM has been identified as being associated with tuberous sclerosis complex (TSC) in its pulmonary manifestation (TSC‐LAM), a multisystem genetic disorder resulting from mutations in either the *TSC1* or *TSC2* genes. Herein, we describe an early 20s female admitted to the hospital with dyspnea, chest pain, hypopigmented macules, and facial fibroadenomas. She has a medical history of renal angiomyolipomas (ALMs) and pneumothoraces. Diagnosis with LAM was confirmed through high‐resolution computed tomography (HRCT) scan and histopathology of lung biopsy. Whole exome sequencing analysis identified a frameshift mutation c.4504del (p.L1502Cfs*74) in the patient's *TSC2* gene. This variant was de novo due to its absence in the patient's parents. This is the first report on the clinical and genetic etiology of TSC‐LAM in Vietnam.

## Graphical abstract

Herein, we describe an early 20s female admitted to the Hospital with dyspnea, chest pain, hypopigmented macules, and facial fibroadenomas. She has a medical history of renal angiomyolipomas (ALM) and pneumothoraces. Diagnosis with LAM was confirmed through high‐resolution computed tomography (HRCT) scan and histopathology of lung biopsy. Whole exome sequencing analysis identified a frameshift mutation c.4504del (p.L1502Cfs*74) in the patient's *TSC2* gene. This variant was de novo due to its absence in the patient's parents. This is the first report on the clinical and genetic etiology of TSC‐LAM in Vietnam.

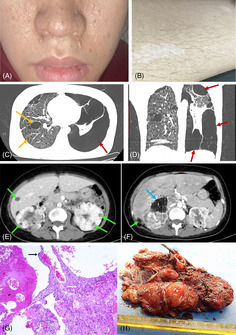

## INTRODUCTION

Pulmonary lymphangioleiomyomatosis (LAM) is a rare, primarily progressive lung disease and affects predominantly women at ‘child‐bearing’ age. In lymphangioleiomyomatosis (LAM), the pathogenesis involves the aberrant proliferation and expansion of atypical smooth muscle‐like cells (LAM cells) within the pulmonary parenchyma, alveolar walls, mediastinum, and lymphatic nodes. This pathological process culminates in the formation of cysts, which systematically compromise pulmonary function by eroding the integrity of healthy lung tissue. The progression of these cysts instigates a cascade of clinical sequelae, including obstruction of the lymphatic system and pleural effusions, manifesting clinically as dyspnea, thoracic discomfort, and respiratory distress. LAM patients were often manifested in pneumothoraces, which appear in around 50%–60% of patients, and dyspnea in 71%–73%.^1^

LAM may manifest sporadically (sporadic‐LAM) or be linked to tuberous sclerosis complex (TSC‐LAM).^2^ Hepatic and renal AMLs, nephrectomy, and noncalcified pulmonary nodules were observed to occur more frequently in patients with TSC‐LAM than normal LAM.^3^

Herein, we provide the first comprehensive report on the clinical and genetic features of a 20‐year‐old female patient with a history of nephrectomy due to renal angiomyolipomas and pneumothorax in Vietnam. Tuberous sclerosis complexes were present via genetic tests, high‐resolution computed tomography (HRCT), and findings of the typical features of pulmonary LAM.

## CASE REPORT

### Clinical finding

**FIGURE 1:**
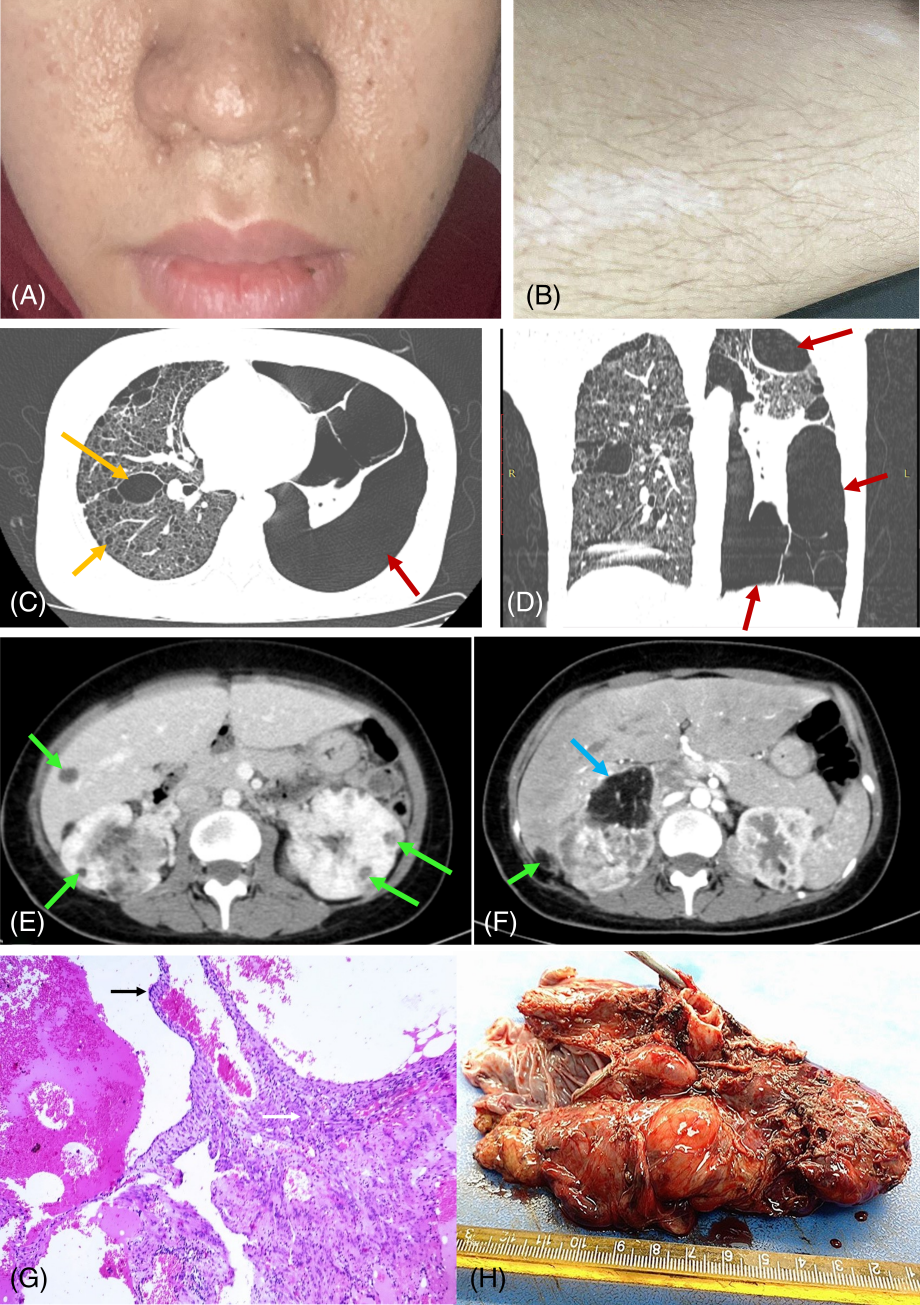
Patient dermal manifestation (A) facial angiofibroma, and (B) two hypomelanotic macules of tuberous sclerosis complex. Chest computed tomography (CT) image of the patient's lungs at different levels and planar (C) axial and (D) coronal. These cysts are seen replacing most of the lung parenchyma (yellow arrow). High‐grade pneumothorax in the left lung due to advanced cystic lung disease (red arrow). (E), (F): Abdomen CT shows multiple enhancing right renal masses, some of which contain macroscopic fat grossly unchanged consistent with angiomyolipomas (green arrow). A portion of the right kidney was surgically resected, resulting in the formation of a fluid‐filled cystic lesion (indicated by the blue arrow). (G) The proliferation of LAM cell nodules with characteristic spindle‐shaped cells (white arrows) in the center and epithelial cells (black arrows) at the periphery (Haematoxylin and Eosin stain at 200x magnification). (H) The diseased lung specimen of the patient was removed in lung transplant surgery.

A 20‐year‐old female experienced dyspnea and chest pain upon admission to the National Lung Hospital (NLH). The patient exhibited facial angiofibroma and hypomelanotic macules in the skin (Figure 1A, B). Her past medical records revealed a laparoscopic nephrectomy performed 8 years prior for the treatment of renal angiomyolipomas. Her respiratory condition worsened following two episodes of pneumothorax approximately 4 years ago.

Pulmonary function tests (PFTs) revealed a restrictive pattern, marked by diminished forced vital capacity (FVC) and forced expiratory volume in 1 s (FEV1), accompanied by an elevated residual volume (RV). The diffusing capacity test (DCT) indicated a significantly reduced diffusion capacity across the alveolar‐capillary membrane. HRCT scans confirmed the presence of diffuse thin‐walled cysts throughout the lungs, a hallmark of LAM, and no nodules were presented (Figure 1C,D). Multiple wide‐spread thin‐walled cysts of variable sizes were observed, some have coalesced into larger cysts in the lung. Abdominal and pelvic CT highlighted renal AMLs containing a mixture of fat and soft tissue (Figure 1E,F). Histopathological examination of the surgical specimen was performed via haematoxylin and eosin (HE) staining (Figure 1G). Based on the history and records, clinical features, and CT examination of the patient, the patient was suspected to have TCS‐LAM.

Given the patient's severe respiratory failure condition, she was added to the awaits list for lung transplant surgery form 2022. Oxygen therapy was used as palliative care for patients since there haven't been any available specific treatments yet. Routine follow‐up check‐ups included pretransplant screening evaluation and managing complications. She underwent a bilateral lung transplant in February 2024. During lung transplant surgery, the LAM lung specimen was resected with a size measuring 18 × 12 × 3.5 cm, which is congested, with adherent fibrous pleura, and has some thin‐walled air sacs at the periphery with  diameters ranging from 2 to 3 cm. There are numerous small nodular cysts inside with diameters ranging from 0.4 to 0.7 cm (Figure 1H).

### Genetic assessment

**FIGURE 2:**
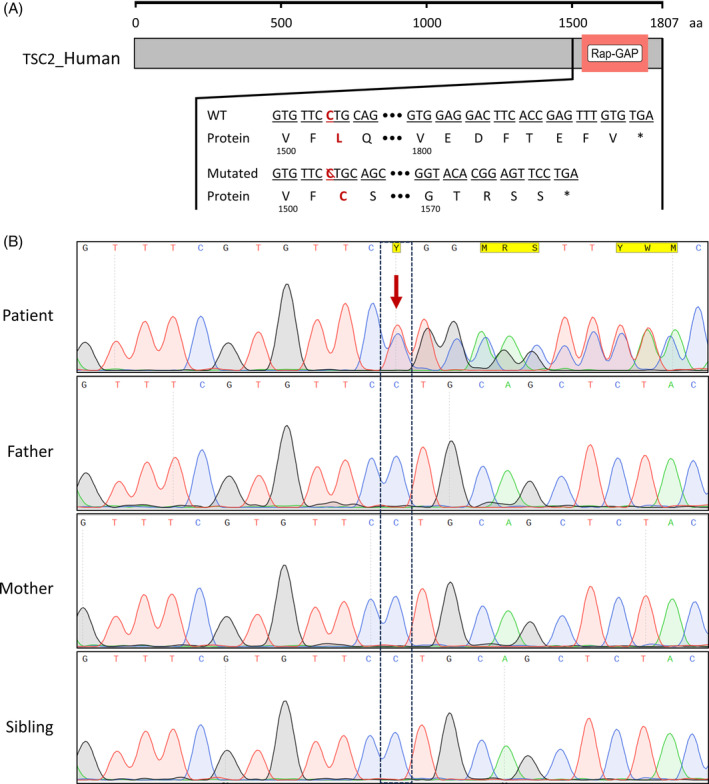
(A) The schematics of TSC2‐human protein (#P49815) with Rap‐GAP domain (pink box) were illustrated. Wild‐type (WT) *TSC2* and mutant with frameshift p.L1502Cfs*74 alternate reading frame ends and code TSC2‐tuberin protein with a truncated Rap‐GAP domain. (B) Sanger chromatogram of p.L1502Cfs*74 mutation (red arrow) presented by heterozygous peaks in proband and absent from family member.

To identify the underlying genetic etiology of the patient's condition, she and her family members were counseled to undergo a genetic examination. The patient and family blood samples were collected for genomic DNA extraction. Initial whole exome sequencing (WES) was performed on the patient's DNA, followed by a screening of variants on ‘Cystic Lung Disease’ genes, including *EFEMP2*, *ELN*, *FBLN5*, *FLCN*, *LTBP4*, *SERPINA1*, *TSC1*, *TSC2*. The analysis identified heterozygous frameshift c.4504del (p.L1502Cfs*74) positioned at the exon 35 of the *TSC2* gene (NM_000548.4), an abridged Rap‐GAP domain at TSC2 protein tail (Figure 2A). The examination of patient's family members by Sanger sequencing showed the absence of the variant *TSC2* c.4504del (p.L1502Cfs*74) in her unaffected parents and sister (Figure 2B). This data suggests that the *TSC2* p.L1502Cfs*74 variant was de novo arose, and resulted in the patient's TSC_LAM condition. The genetic result once again confirmed the diagnosis of TSC‐LAM in the patient.

## DISCUSSION

LAM is the primary pulmonary manifestation affecting postpubescent up to 80% of women with TSC.^2^ A follow‐up on 101 TSC patients has shown the annual rise of LAM was 8% after age 15 and up to 27% before age 21.^2^ The TSC‐LAM condition appears due to inactivating mutations in the *TSC1* or *TSC2* gene, encoding protein tuberin and hamartin, respectively. Mutations disrupt the hamartin‐tuberin complex, a key brake regulator of the mTOR signaling pathway. The TSC1‐TSC2 complex (hamartin‐tuberin) inactivates Rheb‐GTP by activating GTPase thereby leading to the inhibition of the mTOR pathway. Uncontrol activated‐Rheb leads to overactive mTORC1 and a series of proliferation, increased size and survival signals contribute to tumor formation in multi‐organs.^4^ In Vietnam, the first case of LAM in a 36‐year‐old woman was reported in 2022 without being associated with TSC.^5^ Our patient was the first case to present the characteristics of TCS‐LAM, which are consistent with both clinical features and genetic etiology.

The patient presented with dyspnea, chest pain, and reduced capacity air to be inhaled and exhaled forcefully in the lung through PFTs and DCT with a history of two pneumothoraces that are consistent with LAM's clinical features. Multiple rounds, thin‐wall cysts form small to 2 cm scattered diffusely in the right pulmonary cavity, and severe pneumothorax with passive atelectasis in the left cavity were detected in HRCT images. The cysts lined by the relative thin‐wall comprise nodular appearance cells in the patient's lung specimen was visualized in HE straining. The proliferation of the spindle cells underneath the thin‐wall cysts obstructing the lymphatics leads to the formation of numerous cysts. A strong LAM diagnosis was drawn after evaluating all the patient's clinical features through active screening.^1^

Individuals with LAM should undergo a comprehensive assessment of their personal and familial medical history, focusing on the indications of TSC. The examination of patients should encompass an assessment of the skin, retina, and nervous system, taking into account the clinical manifestations associated with TSC.^3^ Patients had medical surgical removal of renal AMLs in 2016 and skin lesion manifestation of angiofibromas and hypomelanotic macules. Renal bilateral ALMs with intratumoral fat components were visualized in the abdominal CT scan indicating recurrence. Transmission of TSC follows an autosomal dominant pattern in the *TSC1* or *TSC2* genes, where instances arise from de novo mutations or inherit the mutation from a parent exhibiting a mild phenotype, potentially remaining undiagnosed. Hence, the inclusion of TSC screening for individuals presenting with LAM significantly influences both patient care and counseling.^3^

In TSC2 protein, the Rap‐GAP catalytic domain acts homology to GTPase‐GTP activity, which plays a crucial role in tumour suppressor towards Rheb.^6^ Due to p.L1502Cfs*74 variant, the Rap‐GAP domain is truncated, leading to a loss of functional integrity. Genetic testing for TSC‐LAM confirmed and enabled the exclusion of familial risk in this case and reformed counseling strategies for patient care. Patients were usually recommended to use Sirolimus, an mTOR inhibitor, which has been demonstrated the potential to prevent recurrent pneumothorax, stabilize lung function, and reduce facial angiofibroma and AMLs.^3^ For cases of severe LAM advancing to end‐stage respiratory failure, lung transplantation emerges as a rescue therapeutic intervention, enhancing both the lifespan and quality of life (73.7% after 10 years, median survival of 12 years).^3^ The recurrent LAM, however, remains a significant concern due to revealed cases of patients' LAM cells with *TSC2* mutation re‐migrate or metastasize back to the lung post‐transplantation.^7^ Our patient just underwent a lung transplant with a positive outcome at the beginning. Her health condition is continuing follow‐up.

This case study underscores the intricate development of LAM under TSC pre‐existent in a young female patient. The genetic etiology was discovered with a de novo frameshift variant, p.L1502Cfs*74, in the *TSC2* gene, leading to curtailment of the Rap‐GAP domain in the tuberin protein. The clinical and genetic findings in this case study highlighted the association between LAM and TSC, and provided valuable insights into the genetic underpinnings of these complex diseases, paving the way for diagnostic and therapeutic strategies.

## AUTHOR CONTRIBUTIONS

All authors discussed the results and contributed to the final manuscript.

## CONFLICT OF INTEREST STATEMENT

None declared.

## ETHICS STATEMENT

The authors affirm securing written informed consent for the publication of this manuscript, along with any accompanying images, ensuring ethical standards are upheld in the publication of the manuscript.

## Data Availability

The data that support the findings of this study are available from the corresponding author upon reasonable request.
